# Anidulafungin dosing during CRRT: do not underestimate adsorption!

**DOI:** 10.1186/s13054-014-0618-6

**Published:** 2014-10-30

**Authors:** Patrick M Honore, Rita Jacobs, Elisabeth De Waele, Herbert D Spapen

**Affiliations:** Intensive Care Department, Universitair Ziekenhuis Brussel, Vrije Universiteit Brussel, 101 Laarbeeklaan, 1090 Brussels, Belgium

Two recent papers in *Critical Care* discuss the use of echinocandins in general [1], and anidulafungin in particular [2], during continuous renal replacement therapy (CRRT). Being highly protein bound and predominantly nonrenally eliminated drugs, echinocandins are barely removed by convection and thus require no dose adjustment during CRRT.

Based on scarce literature data, González de Molina and colleagues additionally state that echinocandin removal by adsorption to the synthetic surface of hemofilters is unlikely to have clinical relevance [1]. This conclusion is largely driven by the study from Leitner and colleagues, who found similar pharmacokinetics of anidulafungin during continuous venovenous hemofiltration in healthy adults and in adults with fungal infections [3]. However, Leitner and colleagues also observed a 20 to 25% loss of initial anidulafungin dose by filter adsorption [3]. This loss resulted in lower maximal concentrations of anidulafungin, yet necessitated no dose adaptation. Importantly, CRRT in this study was performed using polysulfone membranes, which are known to be the least adsorptive.

At present, highly adsorptive membranes (for example, AN69 surface-treated and polymethylmethacrylate filters) are increasingly introduced for CRRT treatment in critically ill patients. As shown for other antimicrobial agents [4], highly adsorptive membranes may adsorb a significantly higher amount of anidulafungin, thereby lowering maximal concentrations to a point at which dose adjustment becomes inevitable. Recent studies also suggest slower saturation (and thus enhanced adsorption capacity) within the bulk of highly adsorptive membranes [5]. Therefore, before recommending unadjusted anidulafungin dosing during CRRT, more information about the handling of this drug by highly adsorptive membranes is urgently needed.

## Abbreviations

CRRT: Continuous renal replacement therapy
